# Tract Ninety-One : An Oxford Object Lesson

**Published:** 1891-04-18

**Authors:** 


### The Hospital

A Weekly Journal of

**Science, Medicine, Nursing, and Philanthropy.**

*For Hospitals, Asylums, Infirmaries, Dispensaries, Medical Practitioners, Students, Nurses, and the Charitable Public.*

Vol. X.—No. 238.] [April 18, 1891.

### Tract Ninety-one: An Oxford Object Lesson.

The "Tracts for the Times" up to ninety mainly concerned themselves with church forms and doctrines. Tract ninety-one will make an attempt, from a medical standpoint, to deal with one part of religious life and practice. Most educated Englishmen are willing to allow the claim of Oxford, that it is the natural home of academic culture, of ecclesiastical religion, and of what may be called "the higher humanity." It ought to be both interesting and instructive to put this claim into a scientific crucible, and to see how it works out in actual experiment.

Oxford has a voluntary hospital called the Radcliffe Infirmary. It is the hospital for the city and the county; it is also the medical school for the members of the University; and its Treasurer and principal official is the Rev. Dr. Bright, the well-known Master of University College. This institution ought, under these circumstances, to represent concrete religion, concrete academic culture, and concrete humanity in their highest forms. We invite the Master of University and the heads of other colleges to consider with us the Radcliffe Infirmary as it is, compared with the Radcliffe Infirmary as it ought to be. Last week an article was published in the Hospital written by our Special Commissioner. The writer was not an inexperienced and irresponsible journalist, but a hospital expert, who has devoted the greater part of his life to practical questions relating to the construction and general management of hospitals. Moreover, his impartiality is unquestionable, and his judgment sound.

"The Radcliffe Infirmary is entered by the basement," says our Commissioner. "The entrance is situated in the oldest part of the buildings, which present an altogether neglected and miserable appearance. The fittings and appliances are for the most part of the oldest and worst description, and the age of most of them tends to discourage those who are responsible for the cleanliness and general appearance of the whole place. . . . It would be difficult to find a parallel to the present condition of many parts of the Radcliffe Infirmary amongst the voluntary institutions of this country." We would like to ask the Master of University College whether his own or any of the other colleges will answer to this description; or whether he can find a single church in the city or suburbs of Oxford that will answer to it? Now, the colleges are mere places of general instruction; and the churches are places of special and higher instruction—instruction, that is to say, in religion, and in the Christian religion. But the Infirmary is a place for the practice of religion, and that in one of its very highest departments. It is a place in which not only are the sick healed, but the sorrowing are soothed, and the destitute succoured. Dr. Bright does not need to be reminded that these duties are among the primest obligations, not only of the New Testament, but of the Old; not only of Holy Scripture, but of church inculcation and church practice from the earliest times. We shall not ask so clear a thinker and so honourable a man as Dr. Bright what may be the value of a religion of which the outward accessories of its schools are so much more valued than the essential parts of the institution at which the religion is practised rather than taught. If it be desirable that churches and colleges be beautiful, it is more than desirable, it is essential that hospitals be wholesome, pleasant, and in every way suitable for the work that is done within their walls. Dr. Bright would not say that a spectacled don who could write a treatise on cricket should be compared as a cricketer with Mr. W. G. Grace; nor do we think that the mere teaching of Christianity and the schools in which it is taught are in any way to be preferred to the practical duties of that faith, or to the places were those duties are carried out.

*Sanitas sanitatum, omnia sanitas*, said Lord Beaconsfield. There are few more pregnant sentences in all Hebrew literature than this. But what says our Commissioner of the sanitary condition of the wards of the hospital of which the greatest University in the world is the patron and neighbour, and of which the head of one of that University's greatest colleges is, or ought to be, the chief ruler and providence? He says: "The condition of the wards, so far as the walls, the sanitary fittings, baths, and other appliances are concerned, exhibits an almost total disregard, not only for appearances, but also for sanitary requirements. Thus in the chief surgical ward, which was regarded as a type of better things twenty years ago, the old, original Parian cement remains in its pristine condition, having never been painted from first to last. Many years ago it was proved that Parian cement was one of the most absorbent of materials, and we venture to think that at least half the thickness of the plaster on the walls is now impregnated with impurities of various kinds." Can it be possible that Dr. Bright, even though he be the clerical head of a college, has never heard of antiseptic medicine and surgery? Is Oxford, that most credulous of all Universities, sceptical alone about germs? Is it necessary that a scientific "Grammar of Assent"

shall be written, and that the authorities of the Radcliffe Infirmary shall be driven into a logical corner, in order that they may be compelled to accept the elementary teachings of physical science, and to act upon them? It almost makes one blush to ask these rudimentary questions! How much more should it make Dr. Bright and the other managers of the Oxford Infirmary blush that such questions should need to be asked!

The Christian religion, of which, in its higher developments, Oxford claims to be a teacher of teachers, has always shown an exceeding regard for children. The little child possesses the typical temper of the kingdom of heaven. "We were informed," says our Commissioner, "that in the children's ward, during the last two years, there have been very few visitors to encourage the workers in the ward, or to sympathise with the poor sufferers in the cots. . . . It is surely a sad reflection on the culture of Oxford, and on its civilisation, that the children's ward should occupy so small a portion of the attention of its inhabitants, and that they should practically leave the paid staff without encouragement—nay, without even the little adjuncts which, so far as we have experienced, are to be found in the children's ward in every other town throughout the United Kingdom." There are crowds of young ladies and other leisured people in Oxford: there are hundreds of young men at her colleges whose time is only half occupied. Some of both those classes must find time hang heavily on their hands. And yet the single children's ward in the one hospital of Oxford cannot find either lady or gentlemen visitors, except at rare intervals. The wealth of Oxford, though it be not like that of the City of London, is yet very considerable for a provincial town. But all the wealth of Oxford does not avail to supply the few children that are in its hospital wards with the little additional comforts which are to be found in practically all the other children's wards of this country. What a commentary this on the delicate and devoted attentions which are lavished upon the Greek particles! How superior are the attractions of what Carlyle contemptuously calls "school logic" and "word-chopping" to the living and suffering children who have both bodies and souls to be cared for! Infant baptism may tear Christianity to pieces, but infant agonies and infant deaths seem to excite but small interest in the learned and acute representatives of the Christian faith at Oxford.

Mr. Casaubon, it is clear, is not yet dead. Or, if he be, he has come to resurrection like a corn of wheat, in a hundred soulless, if not fleshless, representatives of Oxford learning and religion. Do we then undervalue learning? Learning is the noblest of all structures if it be made concrete and solid by life and practice. But learning for learning's sake; learning that leads nowhere; learning that deals in hypotheses, and constructs nothing solid, is as vain and vapid as the song of the blackened Margate minstrel, or as the howl of the mindless drunkard as he reels home on a Saturday night. It is a small thing to ask of Oxford that its hospital shall be made a scientific and sanitary model for all the hospitals in the country. The culture of Oxford and its common sense demand this: but above all, that religion, the beauty of which has been so clearly perceived by so many Oxford eyes, and so rarely illustrated by so many Oxford lives. For her own sake Oxford must remember who it was that said: "Inasmuch as ye have done it unto one of the least of these My brethren, ye have done it unto Me."

